# Management of Traumatic Nerve Palsies in Paediatric Supracondylar Humerus Fractures: A Systematic Review

**DOI:** 10.3390/children10121862

**Published:** 2023-11-27

**Authors:** Christy Graff, George Dennis Dounas, Maya Rani Louise Chandra Todd, Jonghoo Sung, Medhir Kumawat

**Affiliations:** 1 The Women’s and Children’s Hospital, North Adelaide, SA 5006, Australiajonghoo.sung@sa.gov.au (J.S.); 2 Faculty of Health and Medical Sciences, University of Adelaide, Adelaide, SA 5005, Australiamedhir.kumawat@student.adelaide.edu.au (M.K.); 3 The Royal Adelaide Hospital, Adelaide, SA 5000, Australia

**Keywords:** fracture, humerus, nerve injury/palsy/palsies, pediatric/paediatric, supracondylar

## Abstract

**Purpose:** Up to 12% of paediatric supracondylar humerus fractures (SCHFs) have an associated traumatic nerve injury. This review aims to summarize the evidence and guide clinicians regarding the timing of investigations and/or surgical interventions for traumatic nerve palsies after this injury. **Methods:** A formal systematic review was undertaken in accordance with the Joanna Briggs Institute (JBI) methodology for systematic reviews and PRISMA guidelines. Manuscripts were reviewed by independent reviewers against the inclusion and exclusion criteria, and data extraction, synthesis, and assessment for methodological quality were undertaken. **Results:** A total of 51 manuscripts were included in the final evaluation, reporting on a total of 510 traumatic nerve palsies in paediatric SCHFs. In this study, 376 nerve palsies recovered without any investigation or intervention over an average time of 19.5 weeks. Comparatively, 37 went back to theatre for exploration beyond the initial treatment due to persistent deficits, at an average time of 4 months. The most common finding at the time of exploration was entrapment of the nerve requiring neurolysis. A total of 27 cases did not achieve full recovery regardless of management. Of the 15 reports of nerve laceration secondary to paediatric SCHFs, 13 were the radial nerve. **Conclusions:** Most paediatric patients who sustain a SCHF with associated traumatic nerve injury will have full recovery. Delayed or no recovery of the nerve palsy should be considered for exploration within four months of the injury; earlier exploration should be considered for radial nerve palsies.

## 1. Introduction

Nerve palsy is a common complication of paediatric supracondylar humerus fractures (SCHFs), affecting approximately 12% of patients [1,2]. The median nerve proper, or its branching anterior interosseous nerve, is the most commonly impaired nerve from extension-type fractures, while the ulnar nerve is the most at risk of injury in flexion-type fractures [1,2,3]. Over 70% of cases of nerve palsies are present pre-operatively [1].

From the literature and clinical opinion, most reported nerve injuries are managed with a ‘watch and wait’ approach, based on the assumption that the nerve injury is a transient neuropraxia, although the exact resolution details are often unclear [4,5,6]. There is currently no clear evidence regarding the timing of investigation, intervention, and recovery.

This systematic review aims to summarize the current evidence and guide clinicians regarding the timing of investigation and/or surgical intervention for traumatic nerve palsies sustained at the time of injury in paediatric SCHFs and compare the outcomes of nerve palsy in this population with surgical intervention compared with expectant management.

## 2. Methods

The review has been conducted in accordance with the Joanna Briggs Institute (JBI) methodology for systematic reviews of effectiveness with reference to the a priori protocol published in the same journal [7,8]. The review has been registered with the International Prospective Register of Systematic Reviews PROSPERO (CRD42019121581).

A comprehensive search strategy was conducted on 7 June 2021 (Supplementary S1). Randomised controlled trials, cohort studies, case series, and case studies published after 1950 were included. The databases searched were Ovid Medline, Embase, and Cochrane Central, as well as a grey literature search using Google Scholar with the first 200 results returned also reviewed. The bibliographies of the accepted manuscripts were reviewed to identify other relevant published research. The search was re-executed on 23 May 2022, due to the longevity of data curation.

The aim of the study was to compare the effectiveness of operative versus expectant management on the recovery of nerve palsies in paediatric supracondylar fractures. The inclusion criteria were papers that included:

- A paediatric patient with;
- An ipsilateral traumatic upper limb nerve palsy after a SCHF;
- With no pre-existing neurological impairment.

Studies were excluded if:

- They did not provide details regarding follow up or the outcome of the traumatic nerve palsy;
- It was not possible from the reporting to separate individual outcomes from large groups of nerve palsies.

Sequential screening of the manuscripts by title, abstract, and full text were performed by two independent reviewers to determine suitability based on the inclusion and exclusion criteria. The results of the final search were reported in accordance with the preferred reporting items for the systematic reviews and meta-analysis (PRISMA) guidelines [9] (Supplementary S2).

Data extraction was performed by two independent reviewers using a prescribed extraction form. Each eligible manuscript underwent critical appraisal and assessment of methodological quality by two independent reviewers using standardized critical appraisal instruments from the Joanna Briggs Institute (JBI) for Systematic Reviews and Research Synthesis (Supplementary S3–S6) [8]. Cohort studies with complete follow up were scored out of eleven, case series out of ten, and case reports out of eight. Cohort studies without confounding factors or incomplete follow up were scored out of ten, and cohort studies without confounding factors and without incomplete follow up were scored out of nine.

Discrepancies between reviewers at all stages were resolved by a senior reviewer. The primary outcome was nerve palsy recovery, ranging from “full recovery” to “no recovery” as a descriptive measure. Secondary outcomes include time to recovery, modality of treatment, use and timing of investigations, findings at operation, and duration of follow up. Data were synthesised in narrative and tabular format. Due to considerable clinical heterogeneity, a meta-analysis was not performed. Where appropriate, frequencies, percentages, and summaries of data were included for analysis.

## 3. Results

**Figure 1:**
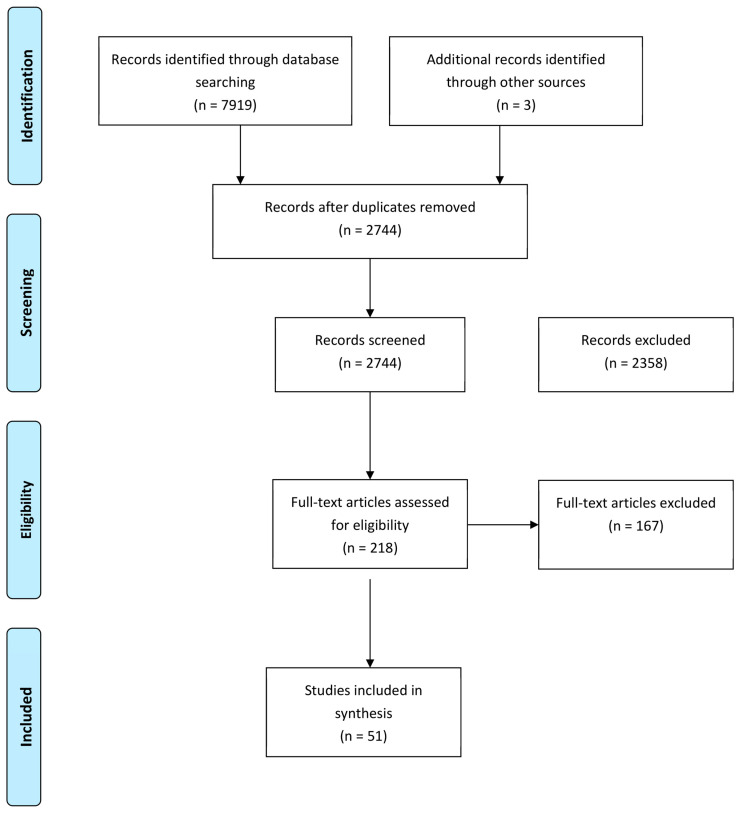
PRISMA flow diagram of the final search.

A total of 7919 results were identified on initial search. All of the results were collated and uploaded into EndNote version X.9 (Clarivate Analytics, Philadelphia, PA, USA) and de-duplication occurred, with a final number of 2744 articles retrieved [10]. After title and abstract screening, there were 218 manuscripts reviewed in full including bibliography reviews, of which 51 met the inclusion criteria and were included in this systematic review as demonstrated in the PRISMA flow diagram (Figure 1) [9]. From the final 51 manuscripts, 16 were case reports/series with the remainder being cohort studies. There were 509 nerve palsies described, with the median nerve most commonly affected and the most common fracture type reported as Gartland type 3. No studies were excluded due to bias (Table 1).

There were 372 traumatic nerve palsies which had full recovery with no intervention (such as nerve exploration) or investigation (such as imaging or nerve conduction studies) undertaken (73.9%) (see Table S1 Supplementary S7). The mean duration of time to full recovery at final follow up in these patients was 19.5 weeks (approximately 5 months) (ranging from 3 days to 1 year). Eight nerve palsies had no intervention (such as nerve exploration) or investigation (such as imaging or nerve conduction studies) and were not fully recovered at last follow up. Davis et al. [21] described an ulnar nerve palsy with sensory disturbance at the 4-year follow up, and two radial nerve palsies with wrist extension weakness at the 4-year follow up. Van Vught et al. [58] reported one patient with ulnar, median, and radial sensory loss after a patient presented to them after 5 days with Volkmann’s ischaemic contracture. Yaokreh et al. [60] reported on two nerves that ‘required electrophysiological studies’ at final follow up but no other detail was given.

There were 26 traumatic nerve palsies which did not document full recovery by the final follow up (5.3%) (see Table S2 Supplementary S7).

There were 92 (18%) nerve palsies which underwent exploration at the time of initial operation (see Table S3 Supplementary S7) of which 89 were described as a secondary intention whilst exploring the brachial artery or an open fracture or converting to open reduction. Three were explored due to surgeon preference of treatment of nerve palsies at presentation [1,19,54]. Eighty-eight which were explored at the time of the initial operation had full recovery by the final follow up, one incomplete recovery, and three were lost to follow up. The findings at exploration in forty-six out of ninety-two nerves were tethered or entrapped in the fracture site, thirty-seven were in continuity, three lacerated, and four contused.

A total of 37 nerves (7.3%) underwent delayed exploration with an average time of 4.4 months (0.5 to 11 months) (see Table S4 Supplementary S7). It was found that 27 were recorded as entrapped in the fracture site/callous/scarring and 10 were found to be completely transected. The radial nerve was involved in sixteen cases, while the median in twelve, and the ulnar in nine. Full recovery at final follow up was reported in 26 nerves. One radial nerve was lacerated, explored, and repaired primarily, but then did not recover, and went on to have a delayed exploration [41]. The primary repair was found to have failed, and was then managed with a nerve graft, and ultimately, tendon transfers.

A total of 13 nerves were found to be completely lacerated on exploration (see Table S5 Supplementary S7). Interestingly, 10 of these were radial nerves. Most of these occurred at the time of the injury, prior to reduction (see Table S5 Supplementary S7).

## 4. Discussion

Our systematic review focused on traumatic nerve palsies in paediatric supracondylar humerus fractures. Iatrogenic or K-wire-associated nerve palsies represent a different spectrum of nerve trauma and have been described elsewhere [30,61]. This review represents the most current comprehensive description of outcomes after traumatic nerve palsies in paediatric supracondylar humerus fractures in the literature, with a total of 510 nerve injuries identified. The characteristics of a Gartland type 3 fracture were consistent with previously reported papers, supporting the opinion that neurological injury is more prevalent amongst more severely displaced fractures [2,24,62]. A total of 18 of the 51 papers did not report the Gartland type, and therefore we did not think a percentage of Gartland type would be accurate to report.

The previous literature reports that 86–100% of nerve injuries will recover spontaneously by 6 months, with a mean time of approximately 3 months [41,63]. Most nerves in the current series were managed expectantly, and had full spontaneous recovery, in keeping with this ‘watch and wait’ policy which is consistently advocated in the literature for patients with anatomical reduction [17,58]. However, an adequate reduction does not rule out the possibility of entrapment and does not account for lacerations [42,55].

A comparison of time frame to full recovery between no exploration, exploration at initial operation, and delayed exploration was unable to be calculated in this review as the majority of papers reported recovery ‘at time of final follow up’ or provided a broad range, such as 1 day to 10 months or 1 to 4 years [17,43]. It is important to recognize that the ‘time to full recovery’ for nerve palsies is the time of final follow up. The literature is not robust enough to determine how long it took for the nerve palsies to fully recover.

It was found that 7.4% of nerve palsies required delayed exploration due to persistent deficits or stagnated recovery at an average of 4 months. Exploration has been advocated for if there is no clinical recovery from 6 weeks to 3 months [30,41,64]. If the nerve is found in continuity at 3 months and is neurolysed, there is a trend to complete recovery [20,58]. Incomplete recovery was more common after complete nerve transection, or if exploration occurred after 4 months. There were 13 reports of nerve laceration secondary to paediatric SCHFs, of which 10 were the radial nerve, and had poorer outcomes.

**Figure 2:**
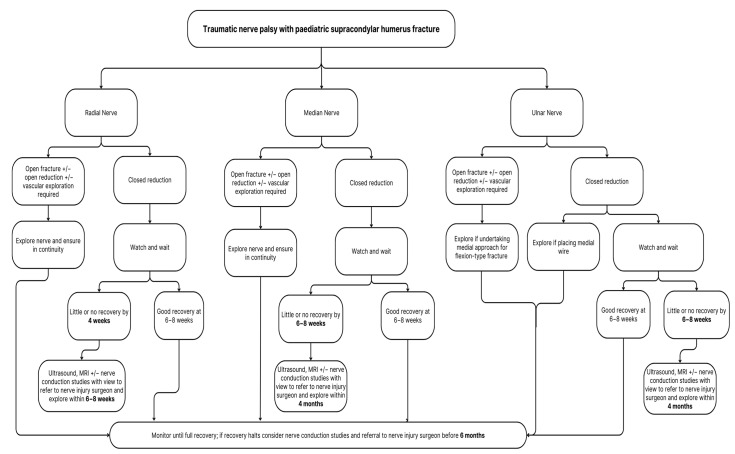
Algorithm for the management of traumatic nerve palsies after paediatric supracondylar humerus fractures.

Nerve exploration is recommended to be undertaken when there is no evidence of clinical or electrophysiological improvement by 8 weeks to 6 months after injury [19,20,45,50,63,65]. Ultrasound has been advocated as useful to evaluate the continuity of the nerve in a small percentage of series pre-operatively, intraoperatively, and post-operatively; ultrasound, however, is highly user dependent [55]. Only three of the papers that met the inclusion criteria reported the use of ultrasound [39,47,55]. Nerve conduction studies and EMG can be poorly tolerated in children, which may explain why most series did not use these in their management of nerve palsies. Magnetic resonance imaging (MRI) can sometimes require a general anaesthetic in this age group but can often be useful in older children to investigate nerve injuries. No study reported on the use of MRI. Additionally, newer surgical techniques such as nerve transfer have not been documented at all in the current literature. From this systematic review, our recommendation would be nerve exploration if there is no or little clinical recovery at 3 months, and exploration within 4 months, except in the case to the radial nerve, which is discussed below. For the consideration of exploration in this time frame, investigations such as ultrasound, MRI, and/or nerve conduction studies should be considered at 6–12 weeks (Figure 2). Liaison with the local nerve injury unit is imperative regarding the timing of referral for the consideration of nerve exploration, repair, nerve grafting, nerve transfer, and/or tendon transfer.

The majority of included papers did not have a primary objective of nerve palsy outcomes; they were commonly reported on only as a complication in part of a wider review of SCHF management techniques. Those that described nerve palsy in detail were often case studies and thus have impacts of selection bias confounding the results. The 5% of nerves that did not fully recover in this series is likely overinflated due to the selection bias of persistent deficits being reported in case series. Our review included eight case reports, focusing only on nerves that were lacerated or entrapped, and so were not typical of the normal pathway of nerve palsies after paediatric supracondylar humerus fractures. There is unfortunately very limited literature dedicated to the management of these injuries from injury to full recovery, which is surprising considering the importance of the topic. Retrospective or prospective data from large centres or multicentre trials on the recovery of nerve palsies in this population is required for improved confidence in recommendations for management.

Another limitation is that two of the largest series describing 425 nerve palsies were excluded by full text; a report of the interventions and outcomes was unclear for the purposes of this review. The authors were contacted for further details which were not able to be obtained at the time of submission [63,66].

The incidence of complete lacerations of nerves after paediatric SCHFs has never been reported before. This systematic review suggests that the radial nerve is more often lacerated than other nerves, which is a new finding to our knowledge. This needs further investigation, and a radial nerve that is not recovering after a paediatric SCHF may need earlier investigation or exploration.

## 5. Conclusions and Recommendations

This is the largest systematic review to report outcomes of investigations and interventions on the recovery of traumatic nerve palsies in paediatric patients after sustaining a supracondylar humerus fracture. From the findings, the authors recommend the below to be included in discussions with parents of these patients:

i. Almost all nerves will fully recover without intervention or investigation within the first 4–5 months;
ii. In nerves with little or no recovery at 3 months, a return to theatre before 4 months is recommended, as full recovery was more likely than those that were not explored, unless the nerve was lacerated;
iii. Although rare, complete transection was reported more commonly in the radial nerve; no recovery of the radial nerve at 6 weeks should alert earlier exploration;
iv. A small percentage (<5%) of traumatic nerve palsies will not fully recover regardless of investigation or surgical exploration; it is likely that permanent damage to the nerve has occurred at the time of fracture, or a failure of the nerve graft or repair.

We encourage centres to report on their outcomes of traumatic nerve palsies after paediatric SCHFs to clarify these recommendations and further guide clinicians.

## Figures and Tables

**Table 1:** Main characteristics of studies included in analysis.

Paper	Study Type	Risk of Bias	No. of Nerve Palsies	Surgical Exploration at ORIF			Delayed Surgical Exploration			Time to Final Follow up in Months
				No	Reason	Findings	No	Timing in Months	Findings	
Ababneh M et al. [11]	RCS	8/10	7	0	n/a	n/a	0	n/a	n/a	6
Aronson DC et al. [12]	RCS	9/11	1	0	n/a	n/a	0	n/a	n/a	12
Ay S et al. [13]	RCS	7/10	9	9	Describing open surgical technique	Median in fracture site ×3, radial kinked ×6	0	0	0	3
Barrett KK et al. [14]	RCS	8/8	35	0	n/a	n/a	0	n/a	n/a	7.4
Bertelli JS & Ghizoni MF [15]	RCS	9/11	6	0	n/a	n/a	6	6–9	Entrapment ×2, laceration ×4	16–24
Boyd DW & Aronson DD [16]	RCS	8/10	3	0	n/a	n/a	0	n/a	n/a	12
Brown IC et al. [5]	RCS	8/9	14	0	n/a	n/a	0	n/a	n/a	6
Campbell CC et al. [17]	RCS	7/9	25	4	3 = VE 1 = FCR	In continuity	0	n/a	n/a	10
Chakrabarti AJ et al. [18]	RCS	7/10	1	1	NE	Complete division	0	n/a	n/a	36
Cheng JC et al. [19]	RCS	7/9	19	1	VE	In continuity	0	n/a	n/a	4–13
Culp RW et al. [20]	RCS	8/9	18	0	n/a	n/a	9	7.5 (mean)	Fibrous scarring ×6, entrapment ×2, laceration ×2	25
Davis RT et al. [21]	RCS	7/9	10	0	n/a	n/a	0	0	0	48
Devkota P et al. [22]	RCS	10/10	6	0	n/a	n/a	0	0	0	3
Dormans JP et al. [23]	CS	5/5	7	0	n/a	n/a	0	0	0	27
Garg Bet al. [24]	CS	8/11	1	1	NE	In continuity	0	n/a	n/a	14–36
Gosens T et al. [25]	RCS	9/11	34	10	VE and FCR	In continuity	0	n/a	n/a	6
Horst M et al. [26]	RCS	8/9	2	0	n/a	n/a	0	n/a	n/a	17 (mean)
Ippolito E et al. [27]	RCS	6/9	14	0	n/a	n/a	1	8	Entrapment in scar tissue	132 (longest)
Jones ET et al. [28]	CS	8/8	6	0	n/a	n/a	0	n/a	n/a	24
Karlsson J et al. [29]	CS	8/8	4	4	VE and FCR	Interposed between bone fragments	0	n/a	n/a	72–108
Khademolhosseini M et al. [30]	RCS	7/9	9	4	FCR	In continuity ×2, entrapment in fracture ×1, contusion ×1	0	n/a	n/a	8
Khan AQ et al. [31]	RCS	8/10	8	0	n/a	n/a	0	n/a	n/a	3
Khan MY et al. [32]	PCaS	8/10	25	2	VE	Entrapment at fracture site	0	n/a	n/a	3
Kirz PH and Marsh HO [33]	CS	8/10	11	0	n/a	n/a	1	4	Lacerated	65
Kiyoshige Y et al. [34]	RCS	7/10	6	0	n/a	n/a	0	n/a	n/a	5–120
Krusche-Mandl I et al. [35]	RCS	8/11	8	0	n/a	n/a	1	2	Compressive scar tissue	12
Kuoppala E et al. [36]	CS	10/10	1	0	n/a	n/a	0	n/a	n/a	12
Lalanandham T et al. [37]	CR	7/8	1	0	n/a	n/a	1	2	Encased in callus; unable to be retrieved	14
Larson AN et al. [38]	RCS	9/11	2	0	n/a	n/a	0	n/a	n/a	10
Leonardi LL et al. [39]	CS	8/10	3	0	n/a	n/a	1	3	Encased at fracture site	12
Li YA et al. [40]	RCS	7/11	7	0	n/a	n/a	0	n/a	n/a	34
Louahem DM et al. [41]	RCS	8/11	66	11	FCR	10 in continuity; 1 radial nerve complete laceration	4	3	Severe compression ×3, laceration and retraction ×1 (lacerated radial nerve failed suture repair)	18
Mangat et al. [42]	CS	10/10	9	5	VE	Entrapment at fracture site	4	1× at 48 h, 2× at 2 weeks, 1× at 3 weeks	Tethered or entrapped	12
Marck KW et al. [43]	CR	8/8	2	2	VE	Laceration ×1, traction ×1	0	n/a	n/a	18–48
Martin DF et al. [44]	CR	8/8	1	0	n/a	n/a	1	6	Laceration	18
McGraw J. et al. [45]	RCS	8/10	17	2	FCR and VE	In continuity	1	6	Laceration	14
Oh CW et al. [46]	RCS	7/10	4	1	VE	Entrapment in fracture	0	n/a	n/a	3
Post M. et al. [47]	CR	8/8	1	0	n/a	n/a	1	6	Encased in callous	30
Rasool MN et al. [48]	CS	8/10	27	27	VE	Kinked ×21, intact ×6	0	n/a	n/a	6
Reigstad O et al. [49]	CS	8/10	2	2	VE	Entrapped in fracture site	0	n/a	n/a	10
Sairyo K et al. [50]	CR	8/8	1	0	n/a	n/a	1	3	Laceration	8
Silva M et al. [51]	RCS	8/11	11	0	n/a	n/a	0	n/a	n/a	6
Solak S et al. [52]	RCS	7/9	6	0	n/a	n/a	0	n/a	n/a	36
Steinman et al. [53]	RCS	8/9	1	1	FCR	Entrapment in fracture site	0	n/a	n/a	1–9
Thorleifsson R et al. [54]	CR	8/8	1	0	n/a	n/a	1	2.5	Entrapment in the fracture site	120
Tokutake et al. [55]	CR	8/8	2	1	NE	Entrapment at the fracture site	1	3	Entrapment at the fracture site	4–6
Tomaszewski et al. [56]	RCS	7/10	22	0	n/a	n/a	2	2	Entrapment at the fracture site	10
Tunku-Naziha TZ et al. [57]	RCS	7/9	2	2	VE	Contused but in continuity	0	n/a	n/a	1.5
van Vugt AB et al. [58]	RCS	7/9	23	1	VE	Complete laceration	0	n/a	n/a	‘Good result’
Yano K et al. [59]	CR	8/8	1	0	n/a	n/a	1	11	Entrapment in callus	36
Yaokreh JB et al. [60]	RCS	7/9	8	0	n/a	n/a	0	n/a	n/a	5–6

RCS = retrospective cohort study; CS = case series; PcaS = prospective case series; CR = case report. VE = vascular exploration; FCR = failed closed reduction; NE = nerve exploration; n/a = Not Applicable.

## Data Availability

Raw data is available on request to the primary author.

## Supplementary Materials

The following supporting information can be downloaded at: https://www.mdpi.com/article/10.3390/children10121862/s1, Supplementary S1: Search Strategies. Supplementary S2: PRISMA 2020 Checklist. Supplementary S3: JBI Critical Appraisal Checklist for Case Control studies. Supplementary S4: JBI Critical Appraisal Checklist for Cohort Studies. Supplementary S5: JBI Critical Appraisal Checklist for Case Series. Supplementary S6: JBI Critical Appraisal Checklist for Case Reports. Supplementary S7: Tables S1–S5. References [67,68,69,70,71,72,73,74] are cited in the supplementary materials.
