# Supplementary material for: Management of Traumatic Nerve Palsies in Paediatric Supracondylar Humerus Fractures: A Systematic Review
Source: Children (Basel). 2023 Nov 27;10(12):1862. doi: 10.3390/children10121862 (PMC10741981; doi:10.3390/children10121862)
Supplement: Supplementary file 1 [file children-10-01862-s001.zip › 20221115 Search strategies 2022 Supplementary S1.pdf]

Supplementary S1

Search strategies run on 7 June 2021

Ovid MEDLINE(R) and Epub Ahead of Print, In-Process, In-Data-Review & Other Non-Indexed Citations and Daily <1946 to June 04, 2021>

1 Humeral Fractures/ 7763

2 ((supracondylar or distal humer* or elbow or gartland) and (fractur* or trauma* or injur*)).ti,ab,kw. 14533

3 1 or 2 19185

4 Peripheral Nerve Injuries/ 7132

5 exp Brachial Plexus/ 24831

6 Brachial Artery/ 10327

7 (anterior interosseous nerve* or AIN or posterious interosseous nerve* or PIN or nerve injur* or nerve palsy or nerve palsies or neuropraxia or median nerve* or brachial arter* or brachial plexus or ulnar nerve* or radial nerve* or neural or neurovascular injur*).ti,ab,kw. 405155

8 exp Neural Conduction/ 32522

9 exp Magnetic Resonance Imaging/ 477282

10 Electromyography/ 80734

11 Nerve Transfer/ 2079

12 Tendon Transfer/ 4243

13 (mri or ultrasound* or nerve conduction stud* or NCS or nerve conduction test* or electromyography or EMG or electrophysiolog* or nerve exploration* or nerve graft* or nerve transfer* or tendon transfer*).ti,ab,kw. 695295

14 4 or 5 or 6 or 7 or 8 or 9 or 10 or 11 or 12 or 13 1400976

15 exp Infant/ 1170773

16 exp Child/ 1973115

17 Adolescent/ 2095394

18 Pediatrics/ 55561

19 (infant* or child* or p?ediatric* or adolescen*).ti,ab,kw. 2105986

20 15 or 16 or 17 or 18 or 19 4224658

21 3 and 14 and 20 2211

22 limit 21 to english language 1846

Embase (Ovid) <1974 to 2021 June 04>

1 humerus fracture/ 9638

2 ((supracondylar or distal humer* or elbow or gartland) and (fractur* or trauma* or injur*)).ti,ab,kw. 17504

3 1 or 2 24839

4 peripheral nerve injury/ 8911

5 exp brachial plexus/ 30697

6 brachial artery/ 15596

7 (anterior interosseous nerve* or AIN or posterious interosseous nerve* or PIN or nerve injur* or nerve palsy or nerve palsies or neuropraxia or median nerve* or brachial arter* or brachial plexus or ulnar nerve* or radial nerve* or neural or neurovascular injur*).ti,ab,kw. 512057

8 exp nerve conduction/ 58643

9 exp nuclear magnetic resonance imaging/ 1031057

10 electromyography/ 81505

11 nerve transplantation/ 4523

12 tendon transfer/ 3979

13 (mri or ultrasound* or nerve conduction stud* or NCS or nerve conduction test* or electromyography or EMG or electrophysiolog* or nerve exploration* or nerve graft* or nerve transfer* or tendon transfer*).ti,ab,kw. 1092729

14 4 or 5 or 6 or 7 or 8 or 9 or 10 or 11 or 12 or 13 2149036

15 exp infant/ 1024152

16 exp child/ 2740054

17 adolescent/ 1589390

18 pediatrics/ 81844

19 (infant* or child* or p?ediatric* or adolescen*).ti,ab,kw. 2584276

20 15 or 16 or 17 or 18 or 19 4214761

21 3 and 14 and 20 2528

22 limit 21 to english language 2208

EBM Reviews - Cochrane Central Register of Controlled Trials (Ovid) <May 2021>

1 Humeral Fractures/ 169

2 ((supracondylar or distal humer* or elbow or gartland) and (fractur* or trauma* or injur*)).ti,ab,kw. 1165

3 1 or 2 1254

4 Peripheral Nerve Injuries/ 103

5 exp Brachial Plexus/ 929

6 Brachial Artery/ 1152

7 (anterior interosseous nerve* or AIN or posterious interosseous nerve* or PIN or nerve injur* or nerve palsy or nerve palsies or neuropraxia or median nerve* or brachial arter* or brachial plexus or ulnar nerve* or radial nerve* or neural or neurovascular injur*).ti,ab,kw. 20091

8 exp Neural Conduction/ 656

9 exp Magnetic Resonance Imaging/ 8748

10 Electromyography/ 3523

11 Nerve Transfer/ 17

12 Tendon Transfer/ 62

13 (mri or ultrasound* or nerve conduction stud* or NCS or nerve conduction test* or electromyography or EMG or electrophysiolog* or nerve exploration* or nerve graft* or nerve transfer* or tendon transfer*).ti,ab,kw. 66266

14 4 or 5 or 6 or 7 or 8 or 9 or 10 or 11 or 12 or 13 88632

15 exp Infant/ 32372

16 exp Child/ 57412

17 Adolescent/ 107543

18 Pediatrics/ 656

19 (infant* or child* or p?ediatric* or adolescen*).ti,ab,kw. 216986

20 15 or 16 or 17 or 18 or 19 303257

21 3 and 14 and 2098

Search strategies updated from 1 June 2021 – 23 May 2022

Ovid MEDLINE(R) and Epub Ahead of Print, In-Process, In-Data-Review & Other Non-Indexed Citations and Daily <1946 to May 20, 2022>

1	Humeral Fractures/	8108
2	((supracondylar or distal humer* or elbow or gartland) and (fractur* or trauma* or injur*)).ti,ab,kw.	15470
3	1 or 2	20254
4	Peripheral Nerve Injuries/	7612
5	exp Brachial Plexus/	25732
6	Brachial Artery/	10617
7	(anterior interosseous nerve* or AIN or posterious interosseous nerve* or PIN or nerve injur* or nerve palsy or nerve palsies or neuropraxia or median nerve* or brachial arter* or brachial plexus or ulnar nerve* or radial nerve* or neural or neurovascular injur*).ti,ab,kw.	437958
8	exp Neural Conduction/	32965
9	exp Magnetic Resonance Imaging/	511448
10	Electromyography/	83175
11	Nerve Transfer/	2348
12	Tendon Transfer/	4428
13	(mri or ultrasound* or nerve conduction stud* or NCS or nerve conduction test* or electromyography or EMG or electrophysiolog* or nerve exploration* or nerve graft* or nerve transfer* or tendon transfer*).ti,ab,kw.	744100
14	or/4-13	1493658
15	exp Infant/	1219468
16	exp Child/	2077323
17	Adolescent/	2176182
18	Pediatrics/	57474
19	(infant* or child* or p?ediatric* or adolescen*).ti,ab,kw.	2219626
20	or/15-19	4395306
21	3 and 14 and 20	2335
22	limit 21 to english language	1966

23 (202106* or 202107* or 202108* or 202109* or 202110* or 202111* or 202112* or 2022*).dt,ez,da. 2188448

24 22 and 23 162

Embase <1974 to 2022 May 20>

1 humerus fracture/ 10005

2 ((supracondylar or distal humer* or elbow or gartland) and (fractur* or trauma* or injur*)).ti,ab,kw. 18232

3 1 or 2 25861

4 peripheral nerve injury/ 9472

5 exp brachial plexus/ 32518

6 brachial artery/ 16275

7 (anterior interosseous nerve* or AIN or posterious interosseous nerve* or PIN or nerve injur* or nerve palsy or nerve palsies or neuropraxia or median nerve* or brachial arter* or brachial plexus or ulnar nerve* or radial nerve* or neural or neurovascular injur*).ti,ab,kw. 538690

8 exp nerve conduction/ 60733

9 exp nuclear magnetic resonance imaging/ 1113911

10 electromyography/ 85783

11 nerve transplantation/ 4779

12 tendon transfer/ 4215

13 (mri or ultrasound* or nerve conduction stud* or NCS or nerve conduction test* or electromyography or EMG or electrophysiolog* or nerve exploration* or nerve graft* or nerve transfer* or tendon transfer*).ti,ab,kw. 1150151

14 or/4-13 2286481

15 exp infant/ 1071565

16 exp child/ 2882743

17 adolescent/ 1669811

18 pediatrics/ 86124

19 (infant* or child* or p?ediatric* or adolescen*).ti,ab,kw. 2732063

20 or/15-19 4424894

21 3 and 14 and 20 2641

22 limit 21 to english language 2319

23 limit 22 to dc=20210601-20220523 203

EBM Reviews - Cochrane Central Register of Controlled Trials <April 2022>

1 Humeral Fractures/ 180

2 ((supracondylar or distal humer* or elbow or gartland) and (fractur* or trauma* or injur*)).ti,ab,kw. 1285

3 1 or 2 1382

4 Peripheral Nerve Injuries/ 105

5 exp Brachial Plexus/ 1031

6 Brachial Artery/ 1232

7 (anterior interosseous nerve* or AIN or posterious interosseous nerve* or PIN or nerve injur* or nerve palsy or nerve palsies or neuropraxia or median nerve* or brachial arter* or brachial plexus or ulnar nerve* or radial nerve* or neural or neurovascular injur*).ti,ab,kw. 21526

8 exp Neural Conduction/ 669

9 exp Magnetic Resonance Imaging/ 9303

10 Electromyography/ 3648

11 Nerve Transfer/ 20

12 Tendon Transfer/ 64

13 (mri or ultrasound* or nerve conduction stud* or NCS or nerve conduction test* or electromyography or EMG or electrophysiolog* or nerve exploration* or nerve graft* or nerve transfer* or tendon transfer*).ti,ab,kw. 70277

14 or/4-13 94015

15 exp Infant/ 34086

16 exp Child/ 60650

17 Adolescent/ 111127

18 Pediatrics/ 681

19	(infant* or child* or p?ediatric* or adolescen*).ti,ab,kw.	226728
20	or/15-19	315275
21	3 and 14 and 20	105
22	limit 21 to yr="2021 -Current"	8
