# Supplementary material for: Management of Traumatic Nerve Palsies in Paediatric Supracondylar Humerus Fractures: A Systematic Review
Source: Children (Basel). 2023 Nov 27;10(12):1862. doi: 10.3390/children10121862 (PMC10741981; doi:10.3390/children10121862)
Supplement: Supplementary file 1 [file children-10-01862-s001.zip › List of Supplementary Digital Content.pdf]

List of Supplementary Digital Content

Supplementary S1: Search Strategies

Supplementary S2: PRISMA 2020 Checklist

Supplementary S3: JBI Critical Appraisal Checklist for Case Control studies

Supplementary S4: JBI Critical Appraisal Checklist for Cohort Studies

Supplementary S5: JBI Critical Appraisal Checklist for Case Series

Supplementary S6: JBI Critical Appraisal Checklist for Case Reports

Supplementary S7: Tables S1–S5
