# Supplementary material for: Management of Traumatic Nerve Palsies in Paediatric Supracondylar Humerus Fractures: A Systematic Review
Source: Children (Basel). 2023 Nov 27;10(12):1862. doi: 10.3390/children10121862 (PMC10741981; doi:10.3390/children10121862)
Supplement: Supplementary file 1 [file children-10-01862-s001.zip › Supplementary S7 Tables S1-S5.pdf]

Supplementary S7 Tables S1-S5

Table S1. All nerve palsies without intervention or investigation.

PAPER	No. of nerve palsies	No. of non-op	Outcomes of non op management
Ababneh M et al. [11]	7	7	FR
Aronson DC et al.[12]	1	1	PR
Barrett KK et al [14]	35	35	FR
Boyd DW & Aronson DD. [16]	3	3	FR
Brown IC et al. [5]	14	14	FR
Campbell CC et al. [17]	25	21	FR
Cheng JC et al. [19]	19	18	FR
Culp RW et al. [20]	18	9	FR
Davis RT et al. [21]	10	10	7 FR<br>3 PR
Devkota P et al. [22]	6	6	FR
Dormans JP et al. [23]	7	7	FR
Gosens T et al. [25]	34	23	FR
Horst M et al. [26]	2	2	FR
Ippolito E et al. [27]	14	12	FR
Jones ET et al. [28]	6	6	FR
Khademolhosseini M et al.[30]	9	5	FR
Khan AQ et al. [31]	8	8	FR
Khan MY et al. [32]	25	23	FR
Kirz PH & Marsh HO[33]	11	10	FR
Kiyoshige Y et al. [34]	6	6	FR
Krusche-Mandl I et al. [35]	8	7	FR
Kuoppala E et al. [36]	1	1	FR
Larson AN et al. [38]	2	2	FR
Leonardi LL et al. [39]	3	2	FR
Li YA et al. [40]	7	7	FR
Louahem DM et al.[41]	66	51	FR
McGraw J. et al [45]	17	13	FR
Oh CW et al. [46]	4	3	FR
Silva M et al. [51]	11	11	FR
Solak S et al. [52]	6	6	FR
Tomaszewski et al. [56]	22	20	FR
van Vugt AB et al. [58]	23	22	FRx19<br>3 = 'bad'
Yaokreh JB et al.[60]	8	8	FRx6<br>PRx2

FR = full recovery, PR = partial recovery.

Table S2. Incomplete recovery at final follow up.

No. of nerves (total)	Median	Radial	Ulnar	Timing of investigation (EMG/NCS/US)	Timing of surgical exploration (months)	Findings at exploration	Outcome at last follow up	Last follow up
1[12]	0	0	1	N/A	N/A	N/A	PR	12 months
3[15]	0	2	0	N/A	6.8	N/A	PR	16-24 months
9 [61]	3	2	4	3 and 5 months	7.5	6 - fibrous scarring<br>2 - Entrapment<br>1 – lacerated (radial)	8 FR<br>1 NR	25 months
1[62]	0	0	1	N/A	at injury	Laceration (ulnar)	PR	3 years
3[63]	0	2	1	N/A	N/A	N/A	PR	4 years
2[27]	0	2	0	8 months and 1 year	8	Entrapped	PR	1 year, 11 years
1[64]	0	1	0	N/A	4	Lacerated (radial)	PR	65 months
1[65]	0	0	1	1 year	2	Entrapment in callous	PR	1 year
2[45]	-	1	1	N/A	6	1x lacerated (radial); other nerve not explored	PR	14 months
3[66]	3	0	0	N/A	3x at injury	Unclear	PR<br>Lost to follow-up	6 months
3[67]	1	1	1	N/A	5 days	Unclear	PR	Unclear
1[68]	1	0	0	11 months	11	Entrapment	PR	3 years
1[69]	1	0	0	3 months	3	Entrapped in callous	PR	4 months
2[60]	Unclear	Unclear	Unclear	N/A	N/a	n/a	Unclear	Unclear
1[70]	1	0	0	3-6 months	Unclear	Entrapment	PR	1 year
1[41]	0	1	0	N/A	At time of fracture and again 3 months	Laceration; repaired Subsequent re-rupture and nerve graft and tendon transfer	PR	5 years

FR= full recovery; PR= partial recovery; NR= no recovery.

Table S3. Surgical Exploration at Primary Treatment.

	Number of nerves explored	Findings	Outcome	Time to last follow up
Ay, Akinci [13]	9	3x median nerves entrapped<br>6x radial nerves contused	FR	3 months
Campbell, Waters [17]	4	In continuity	FR	1 day - 10 months
Chakrabarti and Kunzru [18]	1	Laceration (ulna)	PR	3 years
Cheng, Lam [19]	1	n/a	FR	4-40 weeks
Garg, Weller [1]	1	In continuity	FR	within 36 months
Gosens and Bongers [25]	11	10 in continuity	FR	within 6 months
Karlsson, Thorsteinsson [29]	4	Entrapped	FR	6.75 years (range 6-9)
Khademolhosseini, Abd Rashid [30]	4	1x Ulnar nerve - entrapment; 1xmedian nerve-contused; 1xradial and 1xmedian nerve-in continuity	FR 3.5 months (mean)	8 months
Khan, Younas [32]	2	Entrapment	FR	3 months
Louahem, Nebunescu [41]	11	In continuity; 1xradial nerve laceration	FR except radial nerve	3.3 months (mean)
Mangat, Martin [42]	5	Entrapment	FR	12 months

Marck, Kooiman [43]	2	1x Laceration (median), 1x contused	FR	1.5 - 4 years
McGraw, Akbarnia [45]	2	In continuity	FR	2-3.5 months
Oh, Park [46]	1	Entrapment	FR by 12 weeks	22 months
Rasool and Naidoo [48]	27	5x In continuity, 22x contused	4x FR by day 3, ≤3 months in 15 patient, 3x not recovered by 6 months and lost to follow-up	6 months
Reigstad, Thorkildsen [49]	2	Entrapped	FR	6 months
Steinman, Bastrom [53]	1	Entrapped	FR	36 weeks
Tokutake, Okui [55]	1	Entrapped	FR	4 to 6 months
van Vugt, Severijnen [58]	1	Complete laceration (median)	FR	Unclear
Tunku-Naziha, Wan-Yuhana [57]	2	In continuity	FR	6 weeks

FR=full recovery; PR=partial recovery

Table S4. Delayed nerve explorations.

No. of nerves	Time to exploration (months)	Reason for exploration	Findings	Nerves involved	Investigation	Surgery performed	Outcomes
6[15]	6.7	Persistent deficits	2 entrapped<br>4 lacerated	Radial	n/a	Nerve grafting	3 = Full recovery at 19 months; 3 = Partial recovery at 19 months
9[61]	7.5	Persistent deficits	8 entrapment<br>1 laceration (radial)	2 radial<br>3 median<br>4 ulnar	EMG and NCS at 3 and 5 months	8 neurolysis - all recovered; Lacerated radial nerve required a graft	8= full recovery at 8 months<br>1 (graft) = tendon transfers
1[27]	8	Motor improvement to 3 months then static	Entrapped	Radial	EMG 6 months	Neurolysis	No change over 1 year
1[64]	4	Persistent deficits	Lacerated	Radial	N/A	Primary anastomosis	65 months; motor returned but persistent sensation deficits
1[35]	2	Unclear	Entrapped	Radial	n/a	Neurolysis	Full recovery 4 weeks after exploration (12 weeks post injury)
1[69]	3	Persistent deficits at 3 months	Entrapped	Median	US and NCS; reduced action potentials and loss of continuity	Neurolysis	Improved function at 4 months
1[65]	2	Persistent deficits	Entrapped	Ulnar	EMG 1 year post exploration	Division and Anastomosis	‘Near complete recovery’ at 1 year

4[41]	3	Persistent deficits at 8 weeks	3 ulnar nerves entrapped, 1 radial nerve lacerated	3 ulnar 1 radial	EMG	Neurolysis, radial nerve grafted as primary repair had failed	Ulnar nerve complete recovery, radial persistent deficits
4[42]	0.5-1	Circulatory impairment and sensory deficits	Entrapmen t in fracture site in 3	Median nerve	n/a	Neurolysis	3 = Full recovery at 12 months 1=incomplet e recovery at 12 months
1[44]	6	Persistent deficits	Lacerated	Radial	EMG at 4 - 5 months	Neurolysis and sural nerve graft.	Full recovery by 1 year.
1[45]	6	Persistent deficits	Lacerated	Radial	n/a	Primary repair	Tendon transfers at 14 months
1[47]	6	Persistent deficits and NCS abnormal	Entrapmen t	Median	6 months NCS	Neurolysis	Full recovery at 2.5 years
1[50]	3	Persistent deficits	Lacerated	Radial	n/a	Primary repair	Full recovery at 5 months
1[54]	2.5	Persistent deficits	Entrapmen t	Median	n/a	Resection and primary repair	'uneventful' full recovery at 10 years
1[55]	3	Persistent deficits	Entrapmen t	Radial	n/a	Neurolysis and sural nerve grafting	Full recovery by 6 months
2[6]	1-2	Unclear	Entrapmen t	Median and Ulnar	n/a	Neurolysis	Total group full recovery 122 days (mean)
1[68]	11	Persistent deficits	Entrapmen t	Median	11 months EMG	Neurolysis	Incomplete recovery at 2 years

NCS = nerve conductions studies, EMG = electromyography, US = ultrasound.

Table S5. Nerve lacerations.

Number of nerves (Total)	Type of paper	Nerve	Time to exploration	Investigations (NCS/US/EMG)	Surgery performed	Outcome	Time to last follow up	Pre or post reduction nerve palsy
Chakrabarti and Kunzru [62]	RCS	1 ulnar	Immediate	Nil	Primary nerve repair	PR	3 years	Pre reduction
Marck, Kooiman [43]	CR	1 Median	Immediate (open fracture)	Nil	Primary nerve repair	PR	1.5 - 4 years	Pre reduction
van Vugt, Severijnen [58]	RCS	1 median	? 5 days - unclear	Nil	Primary nerve repair	FR	Unclear	Pre reduction
Bertelli, Soldado [15]	RCS	4 Radial	6-9 months	Nil	Nerve graft	2x FR 2x PR	19 months	No documentation
Culp, Osterman [20]	RCS	1 Radial	7.5 months	EMG and NCS at 3 and 5 months	Complete laceration and grafting	NR Tendon transfers	8 months	No documentation
Kirz and Marsh [33]	CS	1 Radial	4 months	Nil	Complete laceration and primary repair	PR	65 months	Post reduction
Louahem, Nebunescu [41]	RCS	1 Radial	Immediate – repaired, then 4 months	EMG at 8 weeks	Immediate primary repair then at 4 months nerve graft due to failed primary repair	NR Tendon transfers	5 years	Pre reduction
Martin, Tolo [44]	CR	1 Radial	6 months	EMG at 4-5 months	Complete laceration and nerve graft	FR	12 months	Pre reduction

McGraw, Akbarnia [45]	RCS	1 Radial	6 months	Nil	Complete laceration and primary repair	NR Tendon transfers	14 months	Pre reduction
Sairyo, Henmi [50]	CR	1 Radial	3 months	Nil	Complete laceration and primary repair	FR	5 months	Pre reduction

RCS = retrospective cohort study; CS = case series; CR = case report.

FR= full recovery; PR= partial recovery; NR = no recovery.
